# Activation of autophagy in rat brain cells following focal cerebral ischemia reperfusion through enhanced expression of Atg1/pULK and LC3

**DOI:** 10.3892/mmr.2015.3850

**Published:** 2015-05-26

**Authors:** JINGWEI YU, CUIFEN BAO, YANRU DONG, XIA LIU

**Affiliations:** 1 Department of Histology and Embryology, Liaoning Medical University, Jinzhou, Liaoning 121001, P.R. China; 2 Key Laboratory of Molecular Cell Biology and New Drug Development, Liaoning Medical University, Jinzhou, Liaoning 121001, P.R. China

**Keywords:** autophagy, focal cerebral ischemia reperfusion injury, Atg1/pULK, LC3

## Abstract

The present study aimed to investigate the activation of Atg1/pULK, and LC3 in the cerebral cortex following focal cerebral ischemia reperfusion (CIR) injury, thereby examining its effect on autophagy in brain cells. Rat CIR models were established using the technique of middle cerebral artery occlusion. The neurological function score, TTC staining and the water content of brain tissue were used to evaluate the CIR model. Levels of autophagy in the brain cells were examined at different time-points following CIR damage using electron microscopy. Immunohistochemistry and western blot analysis were also used for the qualitative and quantitative detection of levels of Atg1/pULK and LC3 in the cerebral cortex. Autophagy was observed in the early stage of CIR, and the expression of Atg1/pULK and LC3 were observed 1 h following CIR in the rats and reached peak expression levels after12 h, which following which the they gradually decreased. These results suggested Atg1/pULK and LC3 are key in the regulation of autophagy following CIR in the rat brain.

## Introduction

Strokes are characterized by significant rates of mortality and disability (1), and there is increasing interest in elucidating the underlying pathological mechanisms and identifying potential treatment strategies. Previous clinical studies have suggested several effective interventions to improve prognosis (2–4). Following ischemic stroke, the restoration of blood flow is key to tissue repair and functional recovery, however, reperfusion following a period of ischemia may result in cerebral ischemia-reperfusion (CIR) injury. During ischemic injury, several pathological processes are involved, including excitotoxicity, oxidative stress, inflammation and necrotic and apoptotic cell death (5).

The fate of neuronal cells following ischemic stroke is determined by the balance between cell survival and death. Autophagy is regarded as one cell survival mechanism and can be induced by various stress conditions, including oxidative stress and endoplasmic reticulum stress. Following cerebral ischemia and spinal cord injury, enhanced autophagy has been demonstrated (6,7). In different circumstances, autophagy can either prompt cell survival or enhance cell death (8,9). Studies have also indicated that knockdown of Beclin-1 or LC3 significantly suppresses autophagy and enhances cell apoptosis (10,11).

Enhanced autophagy has been identified in cerebral ischemia injury, including global and focal ischemia (12). Following focal CIR, the protein levels of Beclin 1 and LC3 have been found to be significantly upregulated in the post-ischemic brain tissues of rats (13). Transient middle cerebral artery occlusion (MCAO) has been observed to significantly upregulate the numbers of cathepsin D, LAMP1-positive neurons in neonatal rats. In addition, marked punctuate autophagosomal labeling (LC3) and marked lysosomal labeling (cathespin D and LAMP1) are found in the neurons (14). The protein level of cathepsin B is also significantly increased. These results indicate that autophagy is important in neuronal death following focal CIR.

The role of autophagy remains controversial, however increasing studies have indicated consistent autophagy activation following CIR (15,16). The present study aimed to examine the correlation between the expression of Atg1/pULK and LC3 in the cerebral cortex and focal CIR injury, thereby investigating the effect of CIR on autophagy in brain cells.

## Materials and methods

### Animals

A total of 8 adult male Sprague-Dawley rats (220–230 g), provided by the Animal Facility, Health Science Center of Peking University (Beijing, China), were housed in two laboratory animal cages and maintained at 25±1°C with 65±5% humidity on a 12-h light/dark cycle (lights on between 07:30 and 19:30) for at least 1 week prior to experiments. The animals were provided with food and water *ad libitum*. All experimental procedures used in the present study were approved by the Ethics Review Committee for Animal Experimentation of Liaoning Medical University (Jinzhou, China).

### Transient MCAO

The rats were subjected to transient focal cerebral ischemia, induced by right MCAO, as previously described (17), with certain modifications. In brief, the rats were anesthetized with 10% chloral hydrate (360 mg/kg, intraperitioneal), and arterial blood samples, obtained via a femoral catheter, were collected to measure the pO_2_, pCO_2_ and pH using an AVL 998 Blood Gas Analyzer (Roche Diagnostics, Basel, Switzerland). The rectal temperature of the rats was maintained at 37±0.5°C during MCAO via a temperature-regulated heating lamp. A fiber-optic probe was attached to the parietal bone overlying the MCA territory, 5 mm posterior and 5 mm lateral to the bregma, and was connected to a laser-Doppler flowmeter (PeriFlux System 5000; Perimed, Stockholm, Sweden) for continuous monitoring of the cerebral blood flow (CBF). A 40 nylon monofilament suture with a heat-blunted tip was introduced into the internal carotid artery through the stump of the external carotid artery and gently advanced for a distance of 18 mm from the common carotid artery bifurcation to block the origin of the MCA for 90 min, which was then withdrawn to allow reperfusion. Only animals that exhibited a reduction in CBF of >85% during right MCAO, and a CBF recovery of >80% following 10 min of reperfusion were included in the present study. Sham-operated rats underwent the same surgery, however the suture was not inserted. Following closure of the wound, the animals were allowed to recover from anesthesia prior to being returned to their original housing.

### Assessment of neurological deficit score and analysis of survival rates

The neurological deficit score was assessed prior to sacrifice of the rats 24 h after reperfusion, as described previously (18). Each rat was scored by two examiners in a blinded-manner. The following neurological deficit scoring system was used: 0, no motor deficits (normal); 1, forelimb weakness and torso turning to the ipsilateral side when held by tail (mild); 2, circling to the contralateral side but normal posture at rest (moderate); 3, unable to bear weight on the affected side at rest (severe); and 4, no spontaneous locomotor activity or barrel rolling (critical). If no deficit was observed following 2 h recovery post-anesthesia, the animal was excluded from further investigation.

### Edema measurement

A total of 8 rats were sacrificed by decapitation, under deep anesthesia with 10% chloral hydrate, at 6, 12, 24 and 72 h of reperfusion. The ipsilateral and contralateral hemispheres were dissected and the wet weight of the tissues were determined. The tissues were then dried at 120°C for 24 h and the percentage of cerebral water was determined as follows: (wet weight − dry weight) / dry weight × 100.

### Measurement of infarct volume

Following reperfusion, the rats were anesthetized with 3.5% chloral hydrate and then sacrificed by decapitation, following which the whole brains were rapidly removed. Coronal sections (n=10 for each group) were cut into 2 mm slices and stained with standard 2% 2,3,5-triphenyltetrazolium chloride (Sigma-Aldrich, St. Louis, MO, USA) for 10 min at 37°C followed by overnight immersion in 4% formalin. The infarct volume, expressed as a percentage of whole-brain volume, was measured using an image processing and analysis system (Q570IW; 1.25X objective; Leica Microsystems GmbH, Wetzlar, Germany) and was calculated by integration of the infarct area on each brain section along the rostralcaudal axis.

### Immunohistochemistry and immunofluorescence staining

The rats were sacrificed 24 and 72 h after MCAO with an overdose of 3.5% chloral hydrate, and were transcardially perfused with 0.9% saline solution followed by 4% ice-cold phosphate-buffered paraformaldehyde. The brains were then removed, postfixed overnight and equilibrated in phosphate-buffered 30% sucrose. Coronal sections between 1 and 2 mm from the bregma were used, which were cut using a cryostate (Leica CM3000; Leica Microsystems GmbH) at a thickness of 25 mm and used for immunohistochemical staining.

The sections were preserved in liquid nitrogen for 1 week then double-stained by phenotypic markers, using the following primary antibodies: Rabbit polyclonal anti-autophagy LC3 antibody (cat. no. AP1802a; 1:100; Abgent, San Diego, CA, USA) and rabbit poly-clonal anti-Beclin-1 antibody (cat. no. AJ1087a; 1:100; Cell Signaling Technology, Inc., Boston, MA, USA) to label autophagy. The neuronal nuclei (NeuN) neuronal marker, rabbit monoclonal (cat. no. EPR12763; 1:100, Abcam, Cambridge, UK), was used as the internal control. The following secondary antibodies were used: Anti-rabbit and mouse immunoglobulin (Ig) G-fluorescein isothiocyanate and IgG-Cy3 (1:200; Chemicon, Temecula, CA, USA).

### Transmission electron microscopy (TEM)

TEM was used to evaluate ultrastructural changes in the brain sections. Cerebral fragments were fixed with 2.5% glutaraldehyde solution overnight at 4°C; and were then washed with phosphate-buffered saline and fixed with 1% osmic acid for 2 h at room temperature. The tissues were embedded in an epon/araldite mixture (Huntsman Cancer Institute, Salt Lake City, UT, USA) and ultra-thin sections were cut and stained with uranyl acetate (Syntechem Co., Ltd., Changzhou, China) and lead citrate (Xiamen Xingxiang Industrial Co., Ltd., Xiamen, China). The samples were observed under a 1230 type TEM (Japan Electron Optics Laboratory Company, Tokyo, Japan) and images were captured.

### Protein extraction, western blotting and antibodies

Cellular proteins were extracted using radioimmunoprecipitation assay buffer, containing 50 mM Tris/HCl (pH 7.4), 150 mM NaCl 1% (v/v) NP-40 and 0.1% (w/v) SDS (Beijing Solarbio Science and Technology Co., Ltd, Beijing, China), containing 1% (v/v) phenylmethylsulfonyl fluoride (Beijing Solarbio Science and Technology Co., Ltd.), 0.3% (v/v) protease inhibitor (Sigma-Aldrich) and 0.1% (v/v) phosphorylated proteinase inhibitor (Sigma-Aldrich). The lysates were centrifuged at 11,000 × g at 4°C for 15 min and the supernatant was collected to determine the total protein concentration. A bicinchoninic acid protein assay kit (Pierce Biotechnology, Inc., Rockford, IL, USA) was used to determine the protein concentration. Equal quantities of protein (15 *µ*g) were separated on an SDS-PAGE gel (10%; v/v) and transferred onto a polyvinylidene difluoride membrane (Millipore, Darmstadt, Germany). Nonspecific binding was blocked using 8% (w/v) milk in tris-buffered saline and Tween 20 (TBST) for 2 h at room temperature. The membranes were then incubated with primary antibodies against β-Actin (D6A8) (rabbit monoclonal; cat. no. 8457; 1:5,000, Cell Signaling Technology, Boston, MA, USA), ULK1 (D8H5; rabbit monoclonal; cat. no. 8054; 1:1,000, Cell Signaling Technology), Phospho-ULK1 (Ser555; D1H4; rabbit monoclonal; cat. no. 5869; 1:1,000, Cell Signaling Technology), and rabbit polyclonal anti-autophagy LC3 antibody (1:1,000; Abgent, San Diego, CA, USA) overnight at 4°C. Following four washes with TBST, the membranes were incubated with horserasish-peroxidase (HRP)-conjugated goat anti-rabbit and anti-mouse IgG or HRP-conjugated mouse anti-goat IgG (all 1:5,000; Abmart) for 2 h at room temperature and then washed four times. The target proteins were visualized using enhanced chemiluminescence (Millipore Billerica, MA, USA), according to the manufacturer's instructions, and quantified using density analysis normalized against β-actin, according to the manufacturer's instructions, expressed as the fold-change compared with the control.

### Data quantification and statistical analyses

All data are presented as the mean ± standard deviation. Statistical significance was analyzed using a one-way analysis of variance, followed by Tukey's test for multiple comparisons. The t-test was used for comparing the band density values between the groups. P<0.05 was considered to indicate a statistically significant difference.

## Results

### Evaluation of the CIR model in rats

**Figure 1:**
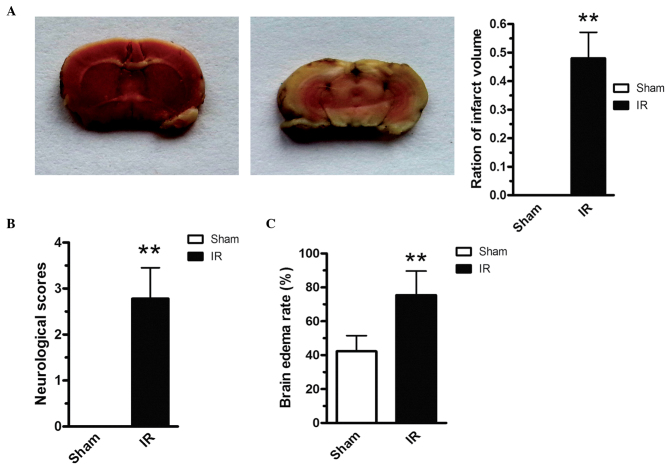
Evaluation of the cerebral infarction post-reperfusion model in rats. (A) Cerebral infarct, (B) neurological deficit scores and (C) brain edema were determined. The quantitative data (n=6 animals per group) are presented as the mean ± standard deviation. ^**^P<0.01 vs. sham group. IR, ischemia reperfusion.

Transient focal ischemia for 2 h caused infarction in the striatum and frontoparietal cortex (Fig. 1A). In addition to the cerebral infarction, 2 h cerebral ischemia also induced severe neurological deficits, compared with the sham-operated rats (Fig. 1B). As shown in Fig. 1C, brain edema was significantly aggregated post-reperfusion. In addition, the water content of the ischemic brain tissues was significantly increased 60 min post-reperfusion (P<0.05; Fig. 1C).

### Involvement of neuronal autophagy following CIR injury

**Figure 2:**
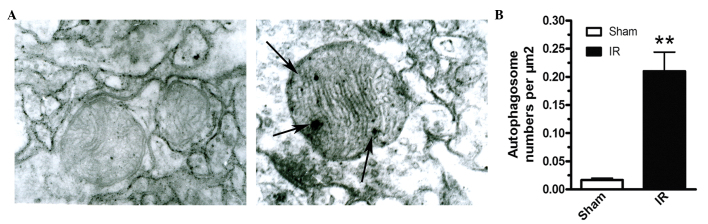
Electron micrographs of morphological changes of cortical neurons after cerebral I/R injury. (A) Broad arrows represent autophagosomes; (B) Quantitative analysis of the numbers of autophagosomes in the IR and sham groups. Three animals were included for each group, with 10 fields in each animal examined. ^**^P<0.01 vs. sham group. IR, ischemia reperfusion.

To evaluate the involvement of neuronal autophagy following CIR injury TEM was used to examine morphological changes in the neurons 60 min following CIR injury. The cortical neurons from the sham-operated control mice maintained a normal appearance of the nuclei, rough endoplasmic reticulum (ER), Golgi apparatus, mitochondria and lysosomes. By contrast, the cortical neurons subjected to CIR exhibited an increase in the number of autophagosomes and autolysosomes. The APs were identified as bubble-like vacuoles in the cytoplasm (Fig. 2).

### Enhanced protein levels of Atg1, pUL and LC3 following CIR injury

**Figure 3:**
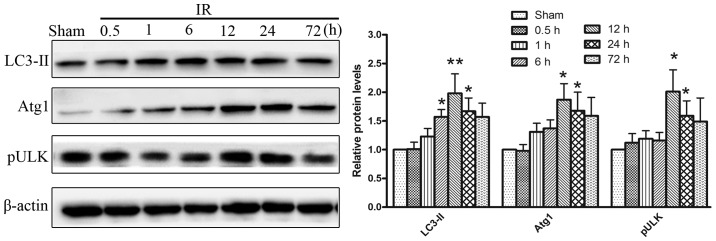
Western blot analysis of autophagy-associated protein expression levels following cerebral IR injury. The levels of Atg1, pULK and LC3 in the ischemic cortex increased significantly between 1 and 24 h reperfusion, with a maximal induction at 12 h. ^*^P<0.05 vs. sham group; ^**^P<0.01 vs. sham group. IR, ischemia reperfusion.

To examine the effect of CIR on autophagy in the brain cells, the levels of autophagy-associated proteins were determined using western blot analysis. Cytoplasmic form LC3 (LC3-I) is diffusely distributed in the cytoplasm. Through modification and conjugation to a phosphatidylethanolamine, the lipidated form (LC3-II) can be attached to the autophago-some membrane during the activation of autophagy, which is a widely accepted marker of autophagy. Atg1/pULK is involved predominantly in the induction of autophagy, and its activity is regulated by the mammalian target of rapamycin (mTOR). As shown in Fig. 3, the expression of LC3-II in the ischemic cortex increased significantly between 1 and 24 h following reperfusion, with a maximal induction at 12 h. The levels of Atg1/pULK were also significantly upregulated and peaked at 12 h.

**Figure 4:**
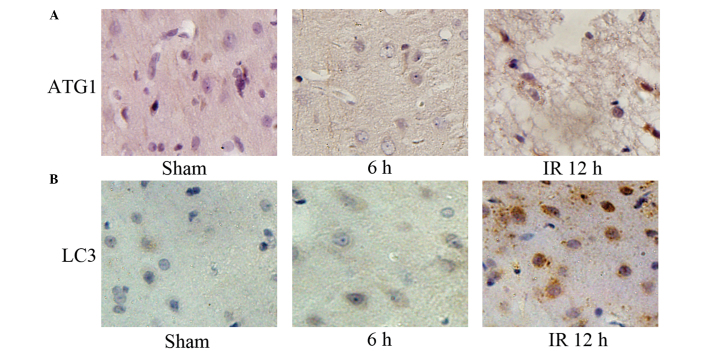
Immunohistochemistry for the expression of LC3 and Atg1 in neurons following cerebral IR injury. (A) In the sham-operated animals, cortical cells exhibited diffuse and weak staining for LC3 in the cytosol. (B) In the sham-operated animals, the cortical cells exhibited significant Atg1 staining 12 h post-reperfusion. ^*^P<0.05 vs. sham group. IR, ischemia reperfusion.

Immunohistochemistry was performed to examine LC3 at 6 and 12 h post-reperfusion. In the sham-operated animals, the cortical cells exhibited diffuse and weak staining for LC3 in the cytosol. Following I/R, intense LC3 staining appeared granular in the cytosol of the cortical cells. Double staining for LC3 and the neuronal nuclei (NeuN) neuronal marker demonstrated increased in LC3 punctate labeling in the cortical neurons, particularly at 12 h (Fig. 4A). Following I/R, the expression of Atg1 increased progressively in size and irregularity (Fig. 4B). Double staining for Atg1 and NeuN revealed that the increased expression of Atg1 occurred predominantly in neurons 12 h post-reperfusion.

## Discussion

The present study provided evidence that autophagy was significantly induced following IR following MCAO in rats. Furthermore, enhanced autophagy was often accompanied by the upregulation of autophagy-associated proteins, including Atg1/pULK and LC3-II in ischemic stroke. These results indicated that autophagy can be markedly induced, in part, by increasing the expression of autophagy-associated proteins.

Ischemic cerebrovascular disease is caused by a blood supply disorder, which is often accompanied by neurologic deficits. Ischemic cerebral vascular diseases account for >60% of the incidence rate of total cerebral vascular diseases (19). According to statistics, ischemic cerebrovascular disease significantly affects human health and quality of life. Therefore, the prevention and treatment of ischemic cerebral vascular disease has become an important topic and has received extensive attention. At present, the predominant treatment of ischemic cerebral vascular disease is to improve blood circulation and remove blood stasis. However, following the restoration of blood supply, the symptoms of certain patients are aggravated, as CIR.

Previous studies have demonstrated that autophagy is important in CIR, however, the mechanism remains to be fully elucidated (6,7,20,21). In the process of autophagy, cytoplasmic components are incorporated into double-membrane vesicles, termed autophagosomes, which can be hydrolyzed by lysosomal hydrolases (22). Autophagy formation can be divided into four stages, which include induction, start, extension and maturity (23). Atg1/pULK is predominantly involved in the induction of autophagy, and its activity is regulated by mTOR (24). Atg6/Belcin 1 is predominantly involved in the initial stage of autophagy. The Vsp34 catalytic subunit of phosphatidylinositol 3-kinase combines with the conserved domain of Atg6/Belcin 1. Autophagy-associated proteins, including Atg21 and Atg24 can then bind to the membrane and form a pre-autophagosomal structure (PAS) (25). In the extension stage, several Atg cytokines are involved in autophagy (26,27). Atg12, Atg5 and Atg16 can form complexes and bind to the PAS; however, Atg8/LC3 is covalently linked to phosphatidylethanolamine, together with Atg7 and Atg3, which then binds to the plasma membrane and is involved in PAS extension. Finally, autophagosomes, namely the autophagic lysosome, can form and, under the action of autophagosomes, the membranes and inclusions can be degraded.

At present, the investigation of autophagy in the model of focal CIR model has been limited to the initial stage of autophagy (Belcin 1). However, the initial and elongation stages have received less attention. The present study focussed predominantly on the regulation factors of these two stages, including Atg1/pULK and LC3. The results of the present study demonstrated significant autophagy initiation 1 h post-CIR. As shown in Figs. 3 and 4, Atg1/pULK were significantly upregulated 1 h post-reperfusion, which peaked at 12 h. In addition, autophagosome formation was also identified 12 h post-reperfusion, with an increase in the number of autophagosomes and autolysosomes observed under TEM. The protein level of LC3-II was also upregulated, suggesting the involvement of autophagy in the cerebral injury post reperfusion.

In conclusion, the present study investigated the expression levels of Atg1/pULK and LC3 in CIR rats, and demonstrated that Atg1/pULK and LC3 were expressed 1 h post-CIR in the rats and reached peak expression at 12 h, prior to subsequent gradual decrease. These results suggested that Atg1/pULK and LC3 are important in the regulation of autophagy following CIR in the rat brain. These data may be useful to further elucidate the mechanism underlying autophagy, thereby providing experimental evidence for the prevention and treatment of CIR.

## References

[1] Hankey GJ, Jamrozik K, Broadhurst RJ, Forbes S, Burvill PW, Anderson CS (2000). Five-year survival after first-ever stroke and related prognostic factors in the Perth community stroke study. Stroke.

[2] Gosman-Hedstrom G, Claesson L, Klingenstierna U, Carlsson J, Olausson B, Frizell M (1998). Effects of acupuncture treatment on daily life activities and quality of life: A controlled, prospective and randomized study of acute stroke patients. Stroke.

[3] Naeser MA, Hamblin MR (2011). Potential for transcranial laser or LED therapy to treat stroke, traumatic brain injury and neurodegenerative disease. Photomed Laser Surg.

[4] Minnerup J, Schabitz WR (2012). Improving outcome after stroke: time to treat new targets. Stroke.

[5] Koike M, Shibata M, Tadakoshi M, Gotoh K, Komatsu M, Waguri S (2008). Inhibition of autophagy prevents hippocampal pyramidal neuron death after hypoxic-ischemic injury. Am J Pathol.

[6] Fan J, Zhang Z, Chao X, Gu J, Cai W, Zhou W (2014). Ischemic preconditioning enhances autophagy but suppresses autophagic cell death in rat spinal neurons following ischemia-reperfusion. Brain Res.

[7] Puyal J, Vaslin A, Mottier V, Clarke PG (2009). Postischemic treatment of neonatal cerebral ischemia should target autophagy. Ann Neurol.

[8] Smith CM, Chen Y, Sullivan ML, Kochanek PM, Clark RS (2011). Autophagy in acute brain injury: Feast, famine, or folly?. Neurobiol Dis.

[9] Puyal J, Ginet V, Grishchuk Y, Truttmann AC, Clarke PG (2012). Neuronal autophagy as a mediator of life and death: Contrasting roles in chronic neurodegenerative and acute neural disorders. Neuroscientist.

[10] Ryter SW, Lee SJ, Smith A, Choi AM (2010). Autophagy in vascular disease. Proc Am Thorac Soc.

[11] Jung G, Roh J, Lee H, Gil M, Yoon DH, Suh C (2015). Autophagic markers BECLIN 1 and LC3 are associated with prognosis of multiple myeloma. Acta Haematol.

[12] Wen YD, Sheng R, Zhang LS, Han R, Zhang X (2008). Neuronal injury in rat model of permanent focal cerebral ischemia is associated with activation of autophagic and lysosomal pathways. Autophagy.

[13] Rami A, Langhagen A, Steiger S (2008). Focal cerebral ischemia induces upregulation of Beclin1 and autophagy-like cell death. Neurobiol Dis.

[14] Puyal J, Vaslin A, Mottier V, Clarke PG (2009). Postischemic treatment of neonatal cerebral ischemia should target autophagy. Ann Neurol.

[15] Zheng YQ, Liu JX, Li XZ, Xu L, Xu YG (2009). RNA interference-mediated downregulation of Beclin1 attenuates cerebral ischemic injury in rats. Acta Pharmocol Sin.

[16] Zhang X, Yan H, Yuan Y, Gao J, Shen Z, Cheng Y (2013). Cerebral ischemia-reperfusion-induced autophagy protects against neuronal injury by mitochondrial clearance. Autophagy.

[17] Hoehn BD, Palmer TD, Steinberg GK (2005). Neurogenesis in rats after focal cerebral ischemia is enhanced by indomethacin. Stroke.

[18] Kofler J, Otsuka T, Zhang Z, Noppens R, Grafe MR, Koh DW (2006). Differential effect of PARP-2 deletion on brain injury after focal and global cerebral ischemia. J Cereb Blood Flow Metab.

[19] Kanno H, Ozawa H, Sekiguchi A, Itoi E (2009). Spinal cord injury induces upregulation of Beclin 1 and promotes autophagic cell death. Neurobiol Dis.

[20] Grishchuk Y, Ginet V, Truttmann AC, Clarke PG, Puyal J (2011). Beclin 1-independent autophagy contributes to apoptosis in cortical neurons. Autophagy.

[21] Rami A, Langhagen A, Steiger S (2008). Focal cerebral ischemia induces upregulation of Beclin 1 and autophagy-like cell death. Neurobiol Dis.

[22] Chen X, Li M, Chen D, Gao W, Guan JL, Komatsu M (2012). Autophagy induced by calcium phosphate precipitates involves endoplasmic reticulum membranes in autophagosome biogenesis. PLoS One.

[23] Koh PO (2008). Melatonin attenuates the focal cerebral ischemic injury by inhibiting the dissociation of pBad from 14-3-3. J Pineal Res.

[24] Lafay-Chebassier C, Paccalin M, Page G, Barc-Pain S, Perault-Pochat MC, Gil R (2005). mTOR/p70S6k signalling alteration by Abeta exposure as well as in APP-PS1 transgenic models and in patients with Alzheimer's disease. J Neurochem.

[25] Meiling-Wesse K, Barth H, Voss C, Eskelinen EL, Epple UD, Thumm M (2004). Atg21 is required for effective recruitment of Atg8 to the preautophagosomal structure during the Cvt pathway. J Biol Chem.

[26] Kofler J, Otsuka T, Zhang Z, Noppens R, Grafe MR, Koh DW (2006). Differential effect of PARP-2 deletion on brain injury after focal and global cerebral ischemia. J Cereb Blood Flow Metab.

[27] Tsubokawa T, Jadhav V, Solaroglu I, Shiokawa Y, Konishi Y, Zhang JH (2007). Lecithinized superoxide dismutase improves outcomes and attenuates focal cerebral ischemic injury via anti-apoptotic mechanisms in rats. Stroke.

